# From Stress Tolerance to Virulence: Recognizing the Roles of Csps in Pathogenicity and Food Contamination

**DOI:** 10.3390/pathogens13010069

**Published:** 2024-01-11

**Authors:** Evieann Cardoza, Harinder Singh

**Affiliations:** Department of Biological Sciences, Sunandan Divatia School of Science, NMIMS University, Vile Parle West, Mumbai 400056, India

**Keywords:** RNA chaperones, cold shock proteins, environmental stress, virulence, stress response, pathogenesis

## Abstract

Be it for lab studies or real-life situations, bacteria are constantly exposed to a myriad of physical or chemical stresses that selectively allow the tolerant to survive and thrive. In response to environmental fluctuations, the expression of cold shock domain family proteins (Csps) significantly increases to counteract and help cells deal with the harmful effects of stresses. Csps are, therefore, considered stress adaptation proteins. The primary functions of Csps include chaperoning nucleic acids and regulating global gene expression. In this review, we focus on the phenotypic effects of Csps in pathogenic bacteria and explore their involvement in bacterial pathogenesis. Current studies of *csp* deletions among pathogenic strains indicate their involvement in motility, host invasion and stress tolerance, proliferation, cell adhesion, and biofilm formation. Through their RNA chaperone activity, Csps regulate virulence-associated genes and thereby contribute to bacterial pathogenicity. Additionally, we outline their involvement in food contamination and discuss how foodborne pathogens utilize the stress tolerance roles of Csps against preservation and sanitation strategies. Furthermore, we highlight how Csps positively and negatively impact pathogens and the host. Overall, Csps are involved in regulatory networks that influence the expression of genes central to stress tolerance and virulence.

## 1. Introduction

The immediate environment of an organism is always changing, and such unstable conditions force it to adapt or succumb to overwhelming pressures. Only those organisms that can detect these changes and mount a defense, neutralize, or tackle the stressor win the race for survival. In the end, these reactions program and reprogram the expression of genes and proteins to protect vital macromolecules and cellular structures. Indeed, the responses displayed by bacteria have caught the attention of researchers and have been the focus of extensive studies to understand the mechanism of adaptation in stressful events. One such stress response that is seen following a drop in temperature is known as a cold shock response and induces a group of proteins called cold-induced proteins (CIPS) [1,2]. Among the CIPS, cold shock proteins (Csps) are a family of small closely related proteins that are widely recognized for their stress tolerance and adaptation roles through their involvement in fine-tuning gene expression as well as transcriptional and translational regulation [3,4]. 

The cold shock domain (CSD) family proteins contain homologous proteins conserved in bacteria [5]. Csps across prokaryotes exhibit a similar β-barrel structure consisting of five antiparallel β-strands and share a high sequence and structural similarity to the other paralogs and homologs. Two of these strands contain evolutionarily conserved RNA-binding motifs—ribonucleoprotein RNP1 and RNP2—that bind and melt nucleic acids [6,7,8,9,10]. A sudden drop in temperature impedes translation and blocks cellular protein synthesis due to the presence of secondary RNA structures that inhibit these processes. The unique cold adaptation property of Csps is attributed to their nucleic acid binding and melting activity that destabilize secondary structures and favors the resumption of growth at low temperatures [10,11,12]. This property is referred to as RNA chaperone activity. Therefore, Csps are also known as RNA chaperones, which are defined as proteins that prevent misfolding or resolve misfolded structures during the process of RNA folding [13]. They are also called RNA-binding proteins (RBPs) as they recognize RNA sequences through their RNA-binding domains (RBDs) [14]. Moreover, cold shock proteins containing a CSD are not solely restricted to prokaryotes but are also identified in eukaryotes with the presence of auxiliary domains in addition to one or more CSDs [15,16,17,18]. Here, they are not confined to the cold shock response but serve pleiotropic functions in the cell [19,20,21,22].

Csps of Gram-positive and Gram-negative bacteria vary in numbers from nine in *Escherichia coli* [23], five in *Bordetella brochiseptica* [24], three in *Bacillus subtilis* and *Listeria monocytogenes* [25,26] to just one in *Thermoanaerobacter tengcongensis* MB4 [27], with a few exceptions being *Mycoplasma* sp, *Helicobacter pylori*, *Chlamydia trachomatis*, *Methanococcus jannaschii*, *Treponema pallidum*, and others which have no Csps [12,28]. A variable number of Csps are, therefore, found in bacteria, and their presence as paralogs can result in overlapping or redundant functions as seen, for instance, in *L. monocytogenes*, which could be an evolutionary backup mechanism [29,30,31]. To complement roles specific to stress tolerance, the presence of functional RNP motifs is crucial. For instance, CspF and CspH of *E. coli* lack conserved aromatic amino acid residues in the RNP motifs and are, therefore, not considered true RNA chaperones. As a result, these proteins are still categorized as uncharacterized proteins, having no specific function assigned to date [5,23,32].

Bacterial Csps were originally discovered in response to a downshift in temperature. However, these proteins are gaining importance for their numerous roles beyond helping the cell adapt to the cold [31,33,34]. Many RBPs and RNA chaperones modulate gene expression, RNA stability, and protein–protein interactions and, accordingly, direct different levels of regulation [14,35,36,37]. Through these mechanisms, CSD family proteins regulate developmental processes in plants [38,39,40], bacterial virulence [30,41], host immune response and infection [42,43,44], as well as bacterial growth at suboptimal and optimal temperatures [45,46]. Therefore, the designation Csps is somewhat of a misnomer as they are also expressed in non-cold stress conditions and facilitate adaptation to diverse sets of stressors [3,47,48,49,50]. 

Csps are currently being looked at from the perspective of regulating virulence-associated genes and mediating pathogenesis within a host [31]. These stress adaptation proteins utilize their ability of stress endurance and offer an upper hand to bacteria in surviving hostile environments within a host. Therefore, Csps are emerging as potential regulatory players in processes concerning pathogenesis. In this review, we first explore how Csps influence genes associated with virulence and influence survival in a host. Next, we address their involvement in food contamination by impacting survival in response to preservation and disinfection strategies. We then, finally, address how these aspects could positively and negatively impact bacteria and the host and discuss future challenges. 

## 2. Cold Shock Proteins in Virulence and Infection

Human pathogens, as well as opportunistic ones, are on the rise and are seemingly difficult to eradicate despite a range of preventive and therapeutic measures [51,52]. In addition, drug-resistant bacteria pose a global threat for which there are no effective antimicrobial therapy for infection control [53]. The infectious potential of bacteria is established by virulence factors, motility, host factors, stress tolerance, as well as capability to form biofilms [54,55,56]. Apart from these well-defined virulence factors, certain proteins regulate virulence-associated gene expression and mediate the process of pathogenesis. The involvement of RNA chaperones and, specifically, cold shock proteins is only recently gaining recognition for their contribution to bacterial pathogenicity. Our understanding of the roles of Csps is centered on stress tolerance and adaptation. This could be one of the reasons that Csps were not observed from the point of view of promoting pathogenicity. Currently, research is focused on investigating the virulent aspects of Csps and their role in aiding the bacterium to survive in harsh environments within a host (Table 1).

### 2.1. Csps Mediate Virulence via Regulation of Stress Tolerance

Previously, bacterial Csps were recognized for their role in transcriptional and translational regulation in response to changing environmental conditions. Such regulation prompts the survival strategy of bacteria in coping with harsh environments. Although the exact mechanisms of Csps within the cell are not clearly defined, these functions are somehow linked to promoting virulence. For instance, many pathogenic bacteria with multiple copies of Csps tend to showcase functions unrelated to cold tolerance by altering gene expression and downstream cellular processes that enhance infectivity. Among them is *Salmonella enterica serovar Typhimurium*, which possesses a family of six copies and requires only two Csps to mediate pathogenicity. Double mutants of CspC and CspE altered responses to stress and virulence, as well as affected host invasion and survival [62]. Deletion mutants and knockout strains have thus provided much clarity on the involvement of Csps in the regulation of virulence-associated genes and pathways. As a case in point, deleting *cspC* in *Acinetobacter baumannii* led to oxidative stress susceptibility and decreased biofilm formation. Upon complementation of the functional Csp, resistance to oxidative stress and virulence was restored as that of the wild type [66]. Along the same lines, the deletion of all three *L. monocytogenes csps* (*ΔcspABD*) resulted in reduced osmotic, cold, and oxidative stress adaptation while also severely impairing intracellular growth in infected macrophages [26,30,63]. 

The route from the environment to a host sees varied surroundings that could be stressful to bacteria. Moreover, once inside a host, the other challenging task is to endure the stresses faced in the host environment. Commensal bacteria are often adapted to fluctuating conditions and are thereby able to persist in complicated surroundings. Any change in the host system could alter the bacterial physiology and direct the shift from normal bacteria to an opportunistic one. Similarly, changes in the host microenvironment were shown to induce stress response genes for survival. For instance, experimental colitis introduced in an IL-10^−/−^ mouse monoassociated with non-pathogenic murine *E. coli* NC101-upregulated bacterial stress response genes including *cspH* and *cspG* [75]. The expression and role of these two Csps in response to intestinal inflammation are yet unclear. However, these stress adaptation behaviors of Csps could potentially be important in surviving the fluctuating conditions particularly observed in a host setting. 

**Figure 1:**
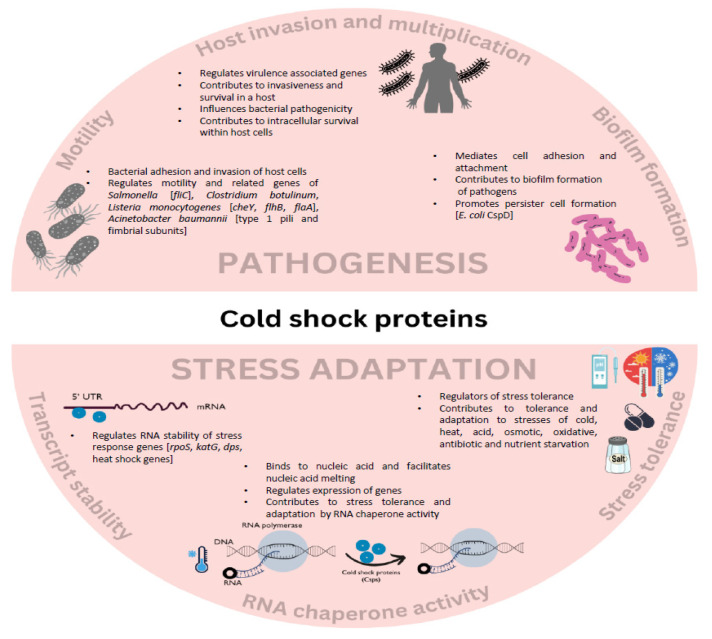
Contribution of Csps in bacterial pathogenesis. The possible molecular mechanisms underlying Csp-mediated virulence are rooted in its chaperone activity. Csps contribute to stress tolerance by increasing mRNA stability and transcript levels, chaperoning structured RNA, and regulating stress gene expression. Csps contribute to bacterial pathogenicity through its regulatory activity by influencing gene expression and pathways related to motility, biofilm formation, host invasion, survival, and proliferation.

The stress adaptation roles of Csps undoubtedly provide them an advantage over survival in harsh backgrounds of low pH and low nutrient availability, as well as the release of reactive oxygen species by the host cells and macrophages. Considering the Csps of *Brucella melitensis* NI, *ΔcspA* mutants were sensitive to the effects of acid and H_2_O_2_, which affected their survival in macrophages. The virulent nature of *B. melitensis* was attenuated in mice in the *cspA* mutant, which led to reduced organ burden as well [59]. The ability of CspA to promote resistance to cold, a low pH of 3.4, and an oxidative challenge with H_2_O_2_ was recovered in the complementing strains, indicating the requirement of CspA in stimulating the virulent nature of *B. melitensis* [59,60]. In a similar manner, when wild-type *Enterococcus faecalis* and its mutant *ΔcspR* strains were tested for their tissue microbial load in a murine systemic infection model, *ΔcspR* mutants exhibited lower bacterial load in the kidneys of infected mice as compared to the control. Additionally, despite the similar phagocytosis of the two strains, *ΔcspR* mutants showed a lower survival in mouse peritoneal macrophages [70]. This could be attributed to the absence of CspR in the mutant that led to reduced stress adaptation in the macrophage as compared to the wild type. Thus, Csps facilitate host invasion and promote stress tolerance responses and proliferation, suggesting important roles in virulence gene regulation (Figure 1). 

### 2.2. Csps Influence Invasiveness of Pathogens

Invasion is one of the initial phases in the establishment of an infection, which is followed by survival and proliferation within a host. The *A. baumannii ΔcspC* mutant significantly reduced survivability in human blood and, when exposed to hydrogen peroxide, simulated the oxidative stress caused by leukocytes upon bloodstream entry [66]. Additionally, mutant strains attenuated pathogenesis in murine infection models through reduced persistence in different organs. Together, these suggest a vital role of CspC in bacterial invasion and the survival of *A. baumannii* in humans. *Enterococcus faecalis* is a Gram-positive and opportunistic pathogen that has an RNA-binding protein CspR required for survival under stationary phase and cold shock [70]. Michaux et al. demonstrated the infectivity of *cspR* mutants and the wild type in the insect infection model of *Galleria mellonella.* Larvae infected with *ΔcspR* mutants had lower mortality rates and showed better survival than those infected with the wild-type *E. faecalis* [70,76]. The virulent nature of *E. faecalis* was restored when complemented with a *cspR* gene encoding a functional CspR, indicating its contribution to the pathogenicity of the opportunistic pathogen within the host.

Given that Csps influence the stability and degradation of mRNA targets, they can control the expression of genes linked to invasion, proliferation, and virulence, which, in turn, allows them to govern pathways related to virulence and pathogenesis [11,48,49,77]. The food-borne pathogen *Listeria monocytogenes* possesses a family of three Csps, namely, CspA, CspB, and CspD, that are required for growth and multiplication in the host environment. A triple deletion mutant of *Listeria* severely compromised intracellular survival in macrophages during infection [30]. In the case of a single functional *csp* gene (double *csp* deletions), a reduced enumeration of intracellular bacteria in macrophages was observed. These studies indicate that the effects of a single Csp cannot restore survival within macrophages, as that of the wild type and *Listeria* requires the expression of the other two *csps* as well [30]. Similarly, the invasive potential of *L. monocytogenes* in Caco-2 and murine macrophage was drastically lowered in single, double, and triple mutant strains except for *ΔcspA* [63]. *ΔcspBD*, and *ΔcspABD* mutants and also significantly impaired invasion after a 12-hour cold adaptation at 4 °C, suggesting that CspB and CspD are required for the host cell invasion of *L. monocytogenes* EGDe. This indicates that certain phenotypes are concealed by functional redundancy among Csps, and this feature could act in favor of pathogens, wherein, if one *csp* is deleted or mutated, its functional role can be substituted by the remaining ones without major loss in viability. 

### 2.3. Csps Regulate Motility-Related Factors and Biofilm Formation

Motility is one such factor that is linked to bacterial invasion and pathogenesis along with other elements like virulence traits, inflammation potential, and microbe load [78,79]. Flagella are crucial components required by pathogenic bacteria because they enable motility and cell attachment [55]. In certain cases, high motility and an increased expression of virulence factors play a role in host invasion and infection [80]. Csps have been reported to regulate virulence factors and, thereby, influence the invasiveness of pathogens. Csps of the *Clostridium botulinum* strain ATCC3502 are involved in the motility of the pathogen, where cells lacking CspB showed reduced mobility at lower temperatures, and mutants of *cspA* and *cspC* hampered flagellation and reduced movement independent of temperature [68]. The role of Csps in the regulation of flagella expression promotes the virulence of pathogenic bacteria as well as their survival within a host and outside host environments [30,61].

Motility-related factors including flagella contribute to virulence, where flagella are associated with bacterial pathogenicity by promoting the initial stages of adherence, motility, biofilm formation, and secretion of virulence proteins into host cells [55,78]. They are directly and indirectly related to biofilm formation by mediating the shift away from stressful environments and enabling surface attachment [78]. Therefore, biofilm and motility often go hand in hand and are frequently linked to virulence by facilitating contact with host cells and establishing themselves in a host [81]. Biofilms bestow upon an organism the ability to cope and evade host-mediated responses, and pathogens with a disposition for biofilm formation are often able to resist adverse conditions and antimicrobial therapies [82,83]. The involvement of Csps in biofilms was reported earlier for *E. coli* CspD, a non-cold inducible Csp that functions as a negative regulator of DNA replication and a nutrient starvation protein. Here, through its interaction with the MqsRA toxin anti-toxin system, it contributes to persister cells and biofilm formation [57,58]. Persister cells are also found in biofilms and are much more resistant to antibiotics. Altogether, bacteria capable of forming biofilms can resist host defenses as well as tolerate treatment with antimicrobials. 

Csps influence pathogenesis by regulating the expression of key genes involved in forming biofilms. As with RNA chaperone activity, Csps are also postulated to interact with DNA, possibly indicating the ability to regulate gene expression. The nucleic acid binding activity was deemed essential for the formation of biofilms and resisting bile, indicating the importance of the chaperone activity of Csps in facilitating pathogenesis [61,84]. Single, double, and triple *csp* deletions of *L. monocytogenes* displayed reduced or a loss of motility, with strains deleted in *cspA* being the major reason for their decrease [30,64]. Additionally, a lower abundance of motility genes in *csp* deletion mutants of *L. monocytogenes* was observed, where low motility *csp* mutants showed an impaired biofilm formation as compared to the motile strains that exhibited better biofilm potential [64]. *ΔcspA* mutants of food and outbreak *L. monocytogenes* strains 568 and 08-5578 also exhibited decreased biofilm formation as compared to their wild types, indicating that CspA is required to form biofilms [64]. Moreover, flagella influence the virulence of pathogens by increasing adherence, colonization, and biofilm formation [78]. In the *ΔcspC* mutant of *A. baumannii*, genes essential for attachment to abiotic surfaces, including type 1 pili and fimbrial subunits, were downregulated. Genes with an overall opposing effect on biofilm production, such as those encoding multidrug efflux pumps, were upregulated, thereby affecting the biofilm formation of the pathogen [66]. Overall, these findings support the role of Csps as regulators of virulence factors. 

Cross-linking and immunoprecipitation methods like RIP-seq or CLIP-seq are often used to identify RNA targets and allow for the mapping of the interactome by directly profiling the RNA that interacts with Csps. Through the use of these techniques, Michaux et al. were able to identify genes in the network of CspC and CspE of *Salmonella.* They reported that these Csps regulate the expression of genes linked to biofilm and motility, such as *fliC*, which codes for flagellin [62]. In addition to this, another study reported reduced transcript levels of class III flagellar genes, namely, *fliC*, *cheY*, *yhjH,* and *motA* in *ΔcspE* mutants [61]. Furthermore, the double mutant *ΔcspCE* caused severe impairment in swimming and swarming motility as well as the attenuation of infection in mice [62]. In a different study, Michaux et al. reported cellular RNA targets of CspC and CspE of an extra-intestinal virulent strain of *E. coli* (ExPEC) involved in serum resistance and virulence [85]. Here, CspC and CspE interacted with several virulence-related transcripts, including *clpX*, *tdcA*, *fur*, and *ryhB*, essential for serum survival and the pathogenesis of ExPEC. 

In conclusion, the role of Csps is important for bacterial virulence through flagella-based motility, surface adherence, cell aggregation, biofilm formation, and survival in the host. Csps enable these virulent phenotypes by regulating mechanisms and controlling gene expression of factors essential to the virulence of pathogens. Together, these signify a direct and crucial involvement of Csps in influencing bacterial pathogenicity.

### 2.4. Csps of Plant Pathogens

Csp-mediated virulence is also recognized in plant pathogens. Here, they play key roles in stress tolerance, enabling phytopathogens to adapt to the stresses and changes experienced by a plant host, as well as regulating the expression of virulence factors and aiding in the establishment of an infection. The *ΔcspD3* mutant of *Ralstonia solanacearum*, for example, reduced the expression of virulence-associated genes, which, in turn, impacted the phytopathogen’s pathogenicity to tobacco. [71]. In a similar case, Csp1 in the phytopathogen *Xylella fastidiosa* strain Stag’s Leap was deemed essential for the survival of the bacterium against stresses of cold and salt [73]. The protein also influenced long-term survival with reduced cell viability as seen in the *csp1*-deficient strain during growth on PD3 agar plates in vitro [72]. Apart from the stress adaptation, Csp1 enabled *X. fastidiosa* to infect susceptible *Vitis vinifera* plants. Here, the mutant’s infectivity was weakened post-inoculation in comparison to the wild type and was regained when complemented with the functional Csp [73]. *Δcsp1* mutants exhibited overall reduced cell-to-cell as well as surface attachment while also being deficient in pili formation [72]. These findings were also confirmed in transcriptomic studies of *Δcsp1* compared with the *X. fastidiosa* wild type, which depicted a downregulation in genes related to biofilm, cell aggregation and attachment, and virulence regulators [72].

*Xanthomonas oryzae* pv. *oryzae* (*Xoo*) PXO99^A^ has four Csps, CspA–CspD, of which CspA contributes to cold adaptation and virulence in rice [74]. CspA affected the biofilm potential along with extracellular polysaccharide (EPS) production, both associated with virulence-related factors in *Xanthomonas*. Transcriptomic and ChIP analysis of *ΔcspA* identified differential expression of genes related to bacterial pathogenicity. Of these, two genes, *PXO_RS11830* and *PXO_RS01060*, were markedly downregulated. Additionally, mutants *ΔPXO_RS11830* displayed impaired biofilm formation, and *ΔPXO_RS01060* reduced EPS production [74]. These findings imply the direct regulation of CspA in virulence against rice and indicate the impact and involvement of Csps in plant pathogenicity.

The precise mechanistic roles of Csps in mediating virulence are currently lacking. Csps are known to influence gene expression and modulate cellular processes due to their chaperone activity [29,62,85]. Here, by maintaining increased transcript stability and regulating the expression of virulence-associated genes, Csps could extend their functions into bacterial pathogenicity. Further investigations are therefore required to understand the exact molecular mechanisms underlying regulation in facilitating pathogenesis. However, it is to be noted that not all Csps are involved in virulence, biofilm formation, and pathogenesis [41,50]. This could indicate that, in certain bacteria, the absence or mutations of specific Csps regulating virulence-associated genes could hamper bacterial survival and pathogenicity within a host. 

There is substantial evidence that bacterial Csps significantly impact genes and proteins connected to virulence, influencing the infectious potential of the bacteria expressing them. In addition to this, the interconnectedness of pathways that Csps partake in tells us that if Csps are mutated or deleted, the pathway function is altered, which, in turn, might be detrimental to the cell in the form of reduced growth or death, unless substituted by another member [62,65]. This indirectly supports functional redundancy in the roles of Csps in pathogenesis.

## 3. Involvement of Csps in Food Contamination

### 3.1. Csps Impact Bacterial Survival under Food Preservation and Disinfection Strategies

The growing food industry is dominated by ready-to-eat meals and preservation strategies that aim to prevent/minimize pathogenic microbial load to avoid food spoilage. Refrigeration for effective storage and minimum food deterioration has been the trend for ages and is a common household technique used in food preservation. To limit microbial growth, food processing units primarily make use of low temperatures to slow down the spoilage of food products and keep microbial growth at bay. This is followed by processes such as drying, the addition of preservatives, and high acid and salt concentrations as preservation techniques [86,87]. Apparently, the preservation methods impart an additional layer of pressure by causing osmotic, oxidative, acidic, and cold stress [88]. Pathogens that can endure these conditions proliferate and can contaminate food. Consequently, considering their survival under stringent preservation and storage settings, getting rid of them now becomes challenging [89].

Low temperatures are often used to extend the shelf-life of food and inhibit bacterial growth. However, Csps are expressed under such conditions and help bacteria adapt and grow, subsequently increasing their chances of contaminating refrigerated food products. Csps are also expressed in response to various stressors, and understanding their involvement in food contamination could contribute to food microbial control measures. Csps regulate genes belonging to global stress systems [10,49,90,91] and help bacteria withstand exposure to pH changes [47], heat [47,48,77,92], high salt concentrations, and low temperatures [26,31,93]. They contribute to bacterial stress tolerance via their RNA chaperone activity and can, therefore, impact bacterial survival and growth under strict conditions of food processing. Furthermore, they extend their regulatory roles to other stresses by offering cross-protection [94,95,96,97]. Therefore, preservation processes that cause a stress response may induce Csps as general stress proteins, potentially increasing the tolerance of foodborne pathogens to stress. This can, subsequently, influence their growth and proliferation, ultimately impacting food spoilage [31,34,98]. This could also increase their ability to grow under higher doses of preservatives. Therefore, when encountered with a new challenge of either low temperatures or high concentrations of preservatives, the adapted bacteria are now primed to endure the new preservation techniques and grow as a contaminant [99]. 

### 3.2. Csps Influence Pathogenesis by Means of Stress Adaptation

*Clostridium botulinum* is a notable food pathogen that can survive high heat due to the presence of spores and produce neurotoxins, ultimately posing a serious health risk. Three Csps, CspA, CspB, and CspC, have been identified in *C. botulinum* ATCC 3502, of which CspB is majorly involved in cold adaptation [69]. In the presence of increasing salt concentrations, strains devoid of CspB and CspC showed reduced growth as observed by longer lag phases. They also demonstrate sensitivity to pH of 5.5 and 6 and lower growth rates to ethanol concentrations of 1–5% [68]. These findings suggest that CspB and CspC could help *C. botulinum* thrive under high doses and stressful conditions of osmotic, acid, and ethanol, which are commonly used preservation and decontamination agents in the food industry. Likewise, higher transcript abundances were reported for *cspD* and *cspA* in late log EGD-e cells of *L. monocytogenes* in BHI supplemented with 3% NaCl [26]. Additionally, single and double mutants of *cspD* in a minimal medium containing 2% NaCl diminished the osmotolerance of *L. monocytogenes*. Therefore, Csps promote growth even under salt stress, suggesting influences of the protein in the regulation of osmotic stress systems. Csps of *L. monocytogenes* also promote tolerance to desiccation and mediate their biofilm potential [64]. Therefore, every step in the preservation and decontamination process could alter the physiology of microbiota already present in food items, be it from plant, animal, or aquatic origin [100]. Consequently, pathogenic bacteria through stress proteins as well as Csps can tolerate and thrive under strict modes of preservation, influence pathogenicity, and, subsequently, impact food contamination. 

The application of sanitizers and disinfectants, either at the growing stage or post-harvest, can prove to be useful in curbing microbial activity associated with food [101,102]. To minimize the microbial load at any stage, treatment with decontaminants such as chlorine, hydrogen peroxide, organic acids, irradiation, and ethanol is undertaken [101,103,104]. When vegetables contaminated with pathogens are consumed raw, it increases the risk of foodborne illness. *E. coli* strains producing verotoxin or Shiga toxin (VTEC/STEC) O157:H7 and non-O157 serotypes have been associated with serious foodborne illness and gastroenteritis. To curb their growth and control food outbreaks, their survival behavior on H_2_O_2_-disinfected lettuce was analyzed [105]. Treatment with 50 mM H_2_O_2_ for 40 min resulted in an upregulation of *cspC* and *cspE* and, to a certain extent, *cspA* in all strains tested [106]. The treatment reduced the microbial load of all VTEC strains on lettuce; however, the same was not observed in the case of pure broth cultures [105,106]. A contrary effect for *cspC* in pure VTEC cultures was observed with the downregulation of the *csp* in the presence of 2.5 mM H_2_O_2_. These studies could indicate the differences in the expression of Csps in pure cultures as opposed to those causing an infection. This could also support the differential nature of Csps in a virulent and non-pathogenic strain, for example, in an extraintestinal pathogenic *E. coli* (ExPEC) as compared to wild-type *E. coli* [47,48,85]. These findings suggest that different environments could affect their activity either towards enhancing their infective potential in a host or solely towards regulating mechanisms contributing to stress tolerance and adaptation.

The transit from a food processing unit to the host exposes bacteria to innumerable backgrounds including changes in temperature and pH. The stresses faced in both these settings are not quite different. Bacteria can survive such conditions with the help of stress proteins and their effects of cross-protection [107]. This would ultimately provide an upper hand to pathogens and increase the risk of foodborne illness. Once inside a host, antibiotics are the main strategy used to eliminate pathogens and eliminate infections. Additionally, when bacteria are exposed to antibiotics, they express Csps, which may help them survive the effect of antibiotics and confer an advantage over other species [24,108,109,110]. This aspect is an additional cause of concern since Csps can influence bacterial survival even when treated with antibiotics.

Foodborne pathogens expressing Csps can, therefore, evade stressful conditions and be a challenge to food safety even under strict preservation strategies. They can contribute to pathogenesis and facilitate the mode from mere stress adaptation to pathogenicity. It is not solely the tolerance and cross-protection but also their participation in virulence that makes Csps candidate proteins to be looked at in terms of food-related microbial control. Therefore, understanding the role of Csps and their participation in pathways promoting tolerance to stressors is crucial and can prove beneficial in the food industry, where microbial contamination at low storage temperatures is a matter of concern. 

## 4. Are Csps the Good or Bad Guys?

Just like every coin has two sides, so too does the activity of Csps. They have the potential to help a bacterium survive harsh surroundings while also being a prospective protein that mediates pathogenicity. Understanding these two aspects could illuminate whether (1) Csps are the good guys for bacteria that help them adapt and survive in the face of a stressful event within a host or (2) the bad guys for the human host, wherein Csps benefit pathogens in facilitating host invasion and infection. 

**Figure 2:**
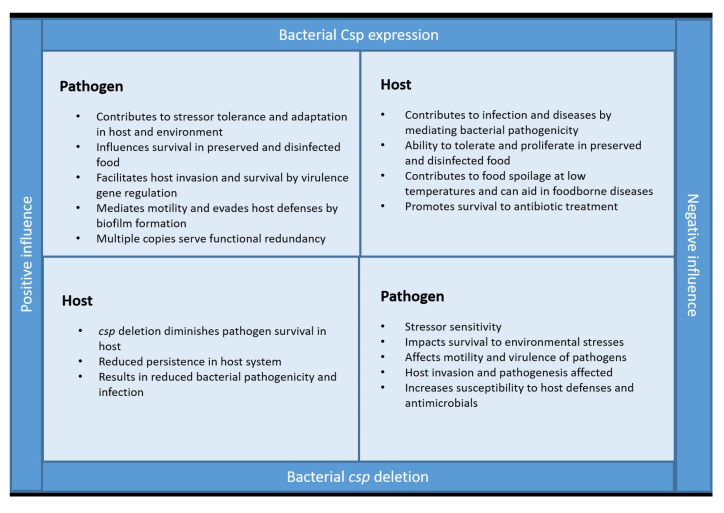
Csps—the good or bad guys? Expression of Csps in pathogens positively influences their survival and proliferation in the environment, on treated food items, and within a host. Csps also contribute to bacterial pathogenicity and give them an upper hand in the host system. On the other hand, bacteria expressing Csps negatively impact the host by increasing the ability of bacteria to promote virulence, food contamination, and infection. However, the *csp* deletion of pathogens acts as a positive factor for the host through their diminished invasion and suppressed tolerance to the stresses usually faced in a host environment. These factors ultimately result in reduced pathogenesis and infectivity. *csp* deletion negatively affects pathogens by weakening virulence and survival in a host. This eventually makes them vulnerable to host defenses. Csps are therefore the good guys for pathogens expressing them and bad guys for the host.

The functional roles of Csps have their pros and cons (Figure 2). Csps behave as a positive factor for bacteria by helping them cope with challenging circumstances. They act on cellular targets in relation to folding, unfolding, and regulating the stability of RNA. In doing so, they aid bacterial species in adapting to stresses and promote survival. Many pathogenic bacteria have multiple copies of Csps that facilitate host invasion, growth, and multiplication, ultimately influencing the infectious potential of the bacterium. Key features of these pathogenesis-associated events involve various proteins in orchestration that contribute to motility, adherence to host cells, survival, and proliferation within a host, ultimately establishing virulence. Csps are regarded as RNA chaperones through specialized motifs that help interact with nucleic acids, and this chaperone activity is deemed non-specific, suggesting that Csps can extend their interaction to a broad range of genes. This is of vital importance to the bacteria under any situation, considering how Csps can bind, interact, and regulate the expression of an extensive set of genes in times of need. Having said this, comprehending the immediate cellular targets and genes associated with Csp-mediated virulence can not only help control certain aspects of host colonization but also uncover the true pathways of Csp-mediated pathogenesis.

When talking about partaking in infections or diseases, bacteria expressing Csps act as a negative factor to the host. Here, they are potentially detrimental given their role of regulating virulence-associated genes while also providing resistance to a multitude of stresses, be it in a host environment or on possessions directly related to humans. In one way or another, this appears to help the organism survive hostile environments in an unfriendly background as well as in a human host. Moreover, the functional redundancy of Csps presents itself as a major disadvantage to the host. Multiple copies in a bacterium could positively impact bacterial pathogenicity through functional complementation. The activity of one Csp when controlled can be substituted by another further complicating matters. 

For the host to have an upper hand, elucidating the immediate targets of Csps and their mechanisms in contributing to virulence could provide a way to curb their activity in vivo. This could hold Csps as targets and help in developing an antimicrobial therapy to limit the occurrence of infection by pathogens. Then again, to understand this facet, exhaustive knowledge of the events of Csp-mediated regulation and its physiological role needs to be implemented from a global perspective. Moreover, deciphering the unique functions of redundant copies in the bacterium could provide substantial evidence of how Csps positively or negatively impact the pathogenesis network through functional complementation. Overall, from their significant participation in pathogenicity, Csps are important regulatory players in the host–pathogen interaction. 

## 5. Future Perspectives

An intriguing avenue for future research lies in unraveling the actual mechanism of action of Csps in virulence factor expression. Moreover, the expression and involvement of Csps differ from strain to strain. Exploring these variations in the roles of Csps among different strains of the same species could shed light on strain-specific responses. Additionally, this could potentially identify key proteins that interact with Csps and ultimately map their regulatory role. These interactions, in the presence of a stressor or a host, could guide our understanding of how they exert virulence and establish pathogenicity. Identifying this could pave the way to target the chaperone networks to curb their action.

Furthermore, investigating the Csps of pathogenic and non-pathogenic bacteria should be explored to clarify their exact role. Two questions need to be addressed at this point: (1) Are Csps of non-pathogenic bacteria specific to stress adaptation? And (2) do Csps of pathogens always contribute to virulence by being commensally suppressed and emerging as virulence-regulating proteins under a specific situation? There is a possibility that Csps behave solely as stress proteins in non-pathogenic bacteria and are capable of contributing to an infection when present in a clinically relevant strain. Does a shift from non-pathogenic/commensal bacteria to pathogenic or an opportunistic one require Csps? Investigating these aspects could prove beneficial in understanding their pleiotropic roles in host invasion and pathogenesis. 

Beyond fundamental research, there is potential for practical applications in clinical settings. Investigating the mechanisms of Csps can contribute to the development of new therapies and antimicrobials. Antibiotics that target bacterial ribosomes have been reported to positively impact the expression of Csps and could be disadvantageous to the host [111,112]. Therefore, antimicrobials that exclusively affect the activity of bacterial Csps should be thoroughly explored. By bridging the gap between basic research and clinical application, we can unlock the translational potential of Csps in addressing challenges related to bacterial virulence and stress response.

## Figures and Tables

**Table 1:** Effects of Csp expression and deletion in stress tolerance and virulence.

Bacterium	Major Disease and Transmission	Csps *	Csp Expression	*Δcsp* Deletion	References
[A] Human pathogens					
*Escherichia coli*	UTI, pneumonia, bacteremia, abdominal and pelvic infection Part of normal microbiota. Transmission by contaminated food	9 CspA-CspI	CspD: induction during starvation and oxidative stress; influences biofilm and persister cell formation	-	[57,58]
*Brucella melitensis*	Brucellosis, zoonosis (contaminated milk products or unpasteurized milk)	4, CspA	Stress responses of acid, cold, oxidative	*ΔcspA* affected metabolism and virulence	[59,60]
*Salmonella typhimurium*	Gastroenteritis. Foodborne, or through contaminated environment	6 CspA-E, CspH	Stress response to cold, oxidative, motility, and biofilm formation	*ΔcspC* and *ΔcspE* altered responses to stress, motility, biofilm, and virulence as well as affected host invasion and survival	[61,62]
*Listeria monocytogenes*	Meningitis and encephalitis. Transmission through contaminated food and mother-to-fetus	3 CspA, CspB, CspD	Nutrient utilization and stress tolerance to cold, osmotic, and oxidative stress.	Deletion of *csps* impairs the utilization of C-sources and compromises cold, pH, and oxidative and osmotic stress tolerance. Mutants show reduced expression of virulence factors, are susceptible to antimicrobials, and are defective in motility, host invasion, and biofilm formation	[26,30,63,64,65]
*Acinetobacter baumannii*	Infection of the lung, blood, wound, and urinary tract. Person-to-person transmission	CspC	-	Hampers biofilm formation, survival, and multiplication in host	[66]
*Staphylococcus aureus*	Bacteremia, infective endocarditis, skin, and bone infections. Person-to-person transmission	3, CspA, CspB, CspC	Stress response to cold	*ΔcspA* upregulated virulence and proteins related to pathogenesis. Downregulated stress response genes, including oxidative stress genes *ΔcspB* shows resistance and susceptibility to certain antimicrobials	[41,67]
*Clostridium botulinum*	Botulism. Transmission through dermal contact and contaminated food	3 CspA, CspB, CspC	Stress response to cold; osmotic	*ΔcspB* and *ΔcspC* are sensitive to low pH, ethanol, and salt	[68,69]
*Acinetobacter oleivorans* DR1	Infection of the lung, blood, wound, and urinary tract. Person-to-person transmission	6	CspA, CspB, CspC, CspE: cold adaptation CspE expression in antibiotic and alkane degradation and downregulation in paraquat and PMS	*ΔcspE* low-temperature growth defect and enhanced biofilm formation	[50]
*Enterococcus faecalis*	Endocarditis, UTI, bacteremia, intra-abdominal, and wound infection Person-to-person transmission	CspR	Cold shock response, stationary phase survival, role in virulence	*ΔcspR* is less virulent than the wild type	[70]
[B] Phytopathogens					
*Ralstonia solanacearum* CQPS-1	Bacterial wilt	4	-	*ΔcspD3* increased swimming motility and decreased virulence-associated genes and virulence potential	[71]
*Xylella fastidiosa*	Bacterial leaf scorch, phony peach disease, Pierce’s disease of grapes, citrus variegated chlorosis	Csp1	Cold and salt stress adaptation	*Δcsp1* impaired cell and surface attachment, biofilm, motility, and virulence	[72,73]
*Xanthomonas oryzae*	Bacterial leaf blight of rice	4, CspA-D	Cold adaptation and virulence	*ΔcspA* affected biofilm and EPS production	[74]

* Column indicates the number of Csp copies and/or Csp members. UTI: urinary tract infection
